# The Delayed Presentation of Bilateral Subdural Hematoma Secondary to Epidural Anesthesia for Elective Vaginal Delivery: A Case Report and Review of the Literature

**DOI:** 10.7759/cureus.59041

**Published:** 2024-04-26

**Authors:** Ahmad Awwad, Rawan A Daraghma, Mohammed M Hajhamad, Diya M Asad, Omar R Khalil

**Affiliations:** 1 Surgery Department, Rafidia Governmental Hospital, Nablus, PSE; 2 Surgery Department, Specialized Arab Hospital, Nablus, PSE; 3 Faculty of Medicine, Al-Quds University, Jerusalem, PSE; 4 Internal Medicine, Al-Quds University, Jerusalem, PSE

**Keywords:** spinal anasthesia, post dural puncture headache, normal vaginal delivery, epidural injections, subdural hemorrhage

## Abstract

Epidural anesthesia (EA) involves reaching the spinal epidural space with an anesthetic drug injection. This procedure provides pain relief during labor. Although EA can lead to some complications, subdural hemorrhage (SDH) is a rare adverse event associated with it. We report the case of a 25-year-old female patient who presented to our emergency department with a one-month history of headaches and associated blurred vision following a normal vaginal delivery with EA. She was initially treated as a case of post-dural puncture headache (PDPH), with no improvement. Finally, the diagnosis of bilateral SDH was made based on a brain MRI. She required surgical intervention, which led to a positive prognosis and a full return of normal baseline neurological functions.

Only a few reports in the literature have indicated the possibility of cranial subdural hematoma formation associated with spinal or epidural analgesia. Our patient experienced a delay in her diagnosis and treatment, as SDH following EA is a rare entity. It is important to follow up with such patients and consider other possibilities when symptoms fail to resolve. Also, reporting these cases is crucial to assist clinicians in early diagnosis and treatment, and to avoid disastrous outcomes.

## Introduction

Epidural anesthesia (EA) was introduced to the medical field in the early period of the 20th century. EA mainly involves introducing a needle to the spinal epidural space and injecting an anesthetic solution into that space. This provides a significant analgesic effect and allows the patient to be fully conscious during the intended procedure for which EA is used [1,2]. Epidural and spinal anesthesia are routine procedures that are currently in wide use in the intrapartum period. A well-known complication of these procedures includes post-dural puncture headaches (PDPH), which may manifest as headaches associated with nausea, vomiting, visual disturbance, or cranial nerve palsies [3].

Cerebrospinal fluid (CSF) leakage through dural puncture causes a reduction in intracranial pressure, leading to the stretching of the bridging veins and pain centers within the brain [4]. Alarming signs that may help differentiate common PDPH from other possible differential diagnoses include persistent headaches not responding to conservative management or postural changes, and the presence of symptoms of neurological dysfunction [5]. Subdural hemorrhage (SDH) forms due to blood accumulation under the dura mater. Our brains have three protective layers: the dura, arachnoid, and pia mater, organized from the outer to the inner parts, respectively [6]. SDH can be classified into three types based on its time of occurrence and radiological appearance. Acute SDH occurs within three days of the causative insult, sub-acute SDH occurs within 4-21 days after the insult, and lastly, chronic SDH occurs 21 days post-insult exposure [7].

Regarding the differences in SDH’s appearance on radiological investigations, acute SDH manifests as a white (hyperdense) collection, sub-acute SDH as gray (isodense), and chronic SDH as black (hypodense) [8]. One of the rarer causes of SDH is EA, with less than 100 cases reported so far and an incidence of one in 500,000 cases [1,9].

## Case presentation

**Figure 1:**
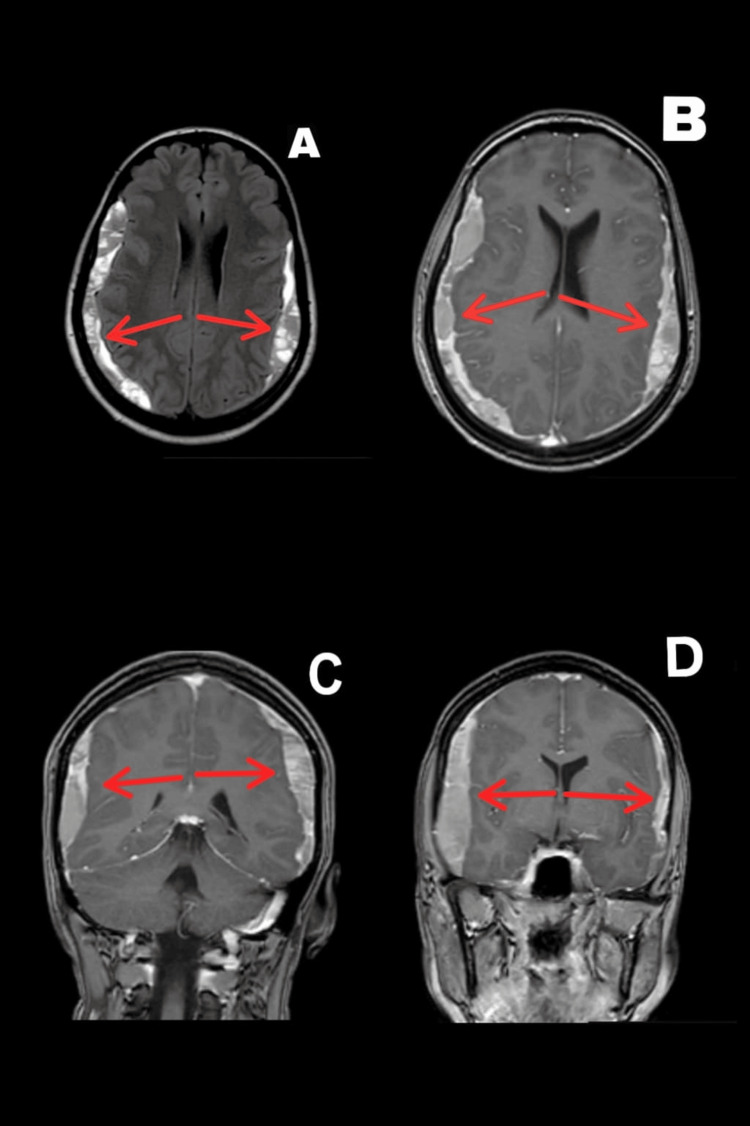
Brain MRI showing bilateral subdural hemorrhage of different ages Figures 1A-1B show the axial view. Figures 1C-1D show the coronal view MRI: magnetic resonance imaging

The patient was a 25-year-old otherwise healthy female patient who underwent a normal vaginal delivery with EA. The procedure was uneventful, and no accidental dural puncture occurred. Two days afterward, she started experiencing headaches with multiple episodes of vomiting. She sought medical advice and was treated as a case of PDPH. The patient had no history of falling or head trauma during that period. Her symptoms did not improve on analgesics and conservative management and progressed to include a progressive blurring of vision in both eyes. The patient’s symptoms persisted despite conservative measures for four weeks. Brain MRI was ordered as a first-line radiological investigation, which showed bilateral SDH of different ages mainly in the sub-acute stage with maximal thickness of 16 mm at the right side and 13 mm at the left side, as illustrated in Figure 1.

Upon arrival at our emergency department, she was conscious, oriented, and alert. She did not suffer from any gross motor or sensory neurological deficits. Fundoscopic examination revealed bilateral severe papilledema. We admitted the patient to the hospital and conducted laboratory investigations including coagulation profiles, which were within normal ranges. We started the patient on anti-seizure prophylaxis in the form of intravenous phenytoin. The patient subsequently underwent a burr-whole craniotomy evacuation and drainage of bilateral SDH with the placement of drains bilaterally.

**Figure 2:**
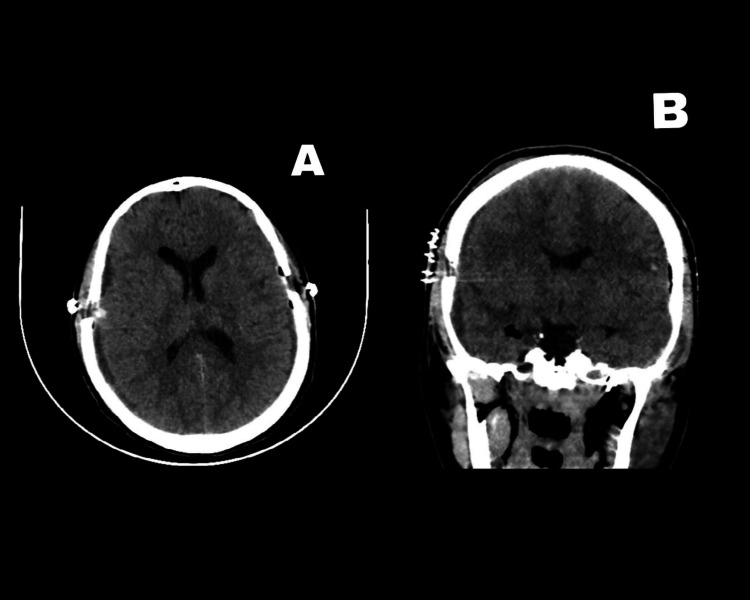
Postoperative head CT scan showing the resolution of the subdural hemorrhage Figure 2A shows the axial view. Figure 2B shows the coronal view CT: computed tomography

Her postoperative period was uneventful; we removed the drains after two days and discharged her a few days later. The postoperative head CT scan showing the resolution of SDH is shown in Figure 2.

**Figure 3:**
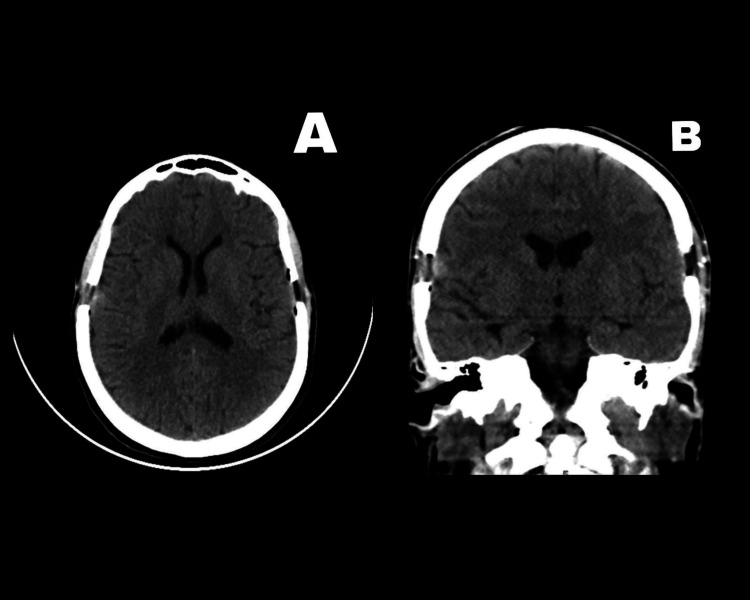
Head CT scan at the follow-up visit showing complete resolution of the hematoma Figure 3A shows the axial view. Figure 3B shows the coronal view CT: computed tomography

We again examined the patient at a follow-up visit three months postoperatively. She was in excellent condition with complete resolution of her previous symptoms. A control head CT scan at the follow-up visit showed the full resolution of the hematoma, as shown in Figure 3.

## Discussion

As mentioned earlier, EA can complicated by SDH in rare cases. It is crucial to maintain a high index of suspicion to adequately diagnose this condition. In our case, the delay in the diagnosis was mainly because this complication did not stand out in the list of differential diagnoses. As such, the potential enduring or life-threatening consequences that might have transpired due to the failure to identify SDH through medical imaging remain a matter of uncertainty. Some cases have reported full recovery with conservative measures [5]. The management of SDH depends mainly on its thickness, midline shifting, and neurological findings. Surgical evacuation of the hematoma is preferred if the clot thickness is more than 10 mm, or if it causes a midline shift of more than 5 mm. Neurological evaluation is pivotal in deciding the method of management if the blood clot’s thickness is less than 10 mm or the midline shifting is below 5 mm [10].

The rate of SDH due to EA may be underrated as some of the cases may be treated conservatively as PDPH. It is only reasonable to consider SDH or even perform an imaging investigation on patients presenting with persistent headaches for more than a week, progressing from postural to non-postural headaches, or in the setting of the emergence of new neurological symptoms [11]. Moreover, while our discussion has centered on SDH as a complication of EA, it is essential to consider alternative diagnoses that may mimic or coexist with SDH, such as PDPH or intracranial hypotension. Distinguishing between these conditions is critical, as each requires distinct management strategies. Therefore, future studies should include a more comprehensive analysis of the differential diagnostic process, emphasizing key clinical features that differentiate SDH from other potential etiologies of persistent headaches following regional anesthesia [11].

## Conclusions

This case report aims to shed more light on some uncommon yet very important medical scenarios. Additionally, it is crucial to compare different management approaches and their outcomes when encountering such scenarios. In our case, SDH was not considered a possible diagnosis, which led to some delay in initiating the management. We strongly recommend considering this possible diagnosis and conducting further research to determine the actual prevalence of this complication in patients who undergo EA.
